# The HRP3 PWWP domain recognizes the minor groove of double-stranded DNA and recruits HRP3 to chromatin

**DOI:** 10.1093/nar/gkz294

**Published:** 2019-04-24

**Authors:** Wei Tian, Peiqiang Yan, Ning Xu, Arghya Chakravorty, Robert Liefke, Qiaoran Xi, Zhanxin Wang

**Affiliations:** 1 Key Laboratory of Cell Proliferation and Regulation Biology of Ministry of Education, College of Life Sciences, Beijing Normal University, 19 Xinjiekouwai Avenue, Beijing 100875, China; 2 MOE Key Laboratory of Protein Sciences, School of Life Sciences, Tsinghua University, Beijing 100084, China; 3 Beijing Advanced Innovation Center for Structural Biology, School of Life Sciences, Tsinghua University, Beijing 100084, China; 4 Department of Physics and Astronomy, Clemson University, Clemson, SC 29634, USA; 5 Institute of Molecular Biology and Tumor Research (IMT), Philipps University of Marburg, Marburg 35043, Germany; 6 Department of Hematology, Oncology and Immunology, University Hospital Giessen and Marburg, 35043 Marburg, Germany

## Abstract

HDGF-related protein 3 (HRP3, also known as HDGFL3) belongs to the family of HDGF-related proteins (HRPs) and plays an essential role in hepatocellular carcinoma pathogenesis. All HRPs have a PWWP domain at the N-terminus that binds both histone and DNA substrates. Despite previous advances in PWWP domains, the molecular basis by which HRP3 interacts with chromatin is unclear. In this study, we solved the crystal structures of the HRP3 PWWP domain in complex with various double-stranded DNAs with/without bound histone peptides. We found that HRP3 PWWP bound to the phosphate backbone of the DNA minor groove and showed a preference for DNA molecules bearing a narrow minor groove width. In addition, HRP3 PWWP preferentially bound to histone peptides bearing the H3K36me3/2 modification. HRP3 PWWP uses two adjacent surfaces to bind both DNA and histone substrates simultaneously, enabling us to generate a model illustrating the recruitment of PWWP to H3K36me3-containing nucleosomes. Cell-based analysis indicated that both DNA and histone binding by the HRP3 PWWP domain is important for HRP3 recruitment to chromatin *in vivo*. Our work establishes that HRP3 PWWP is a new family of minor groove-specific DNA-binding proteins, which improves our understanding of HRP3 and other PWWP domain-containing proteins.

## INTRODUCTION

**Figure 1.**
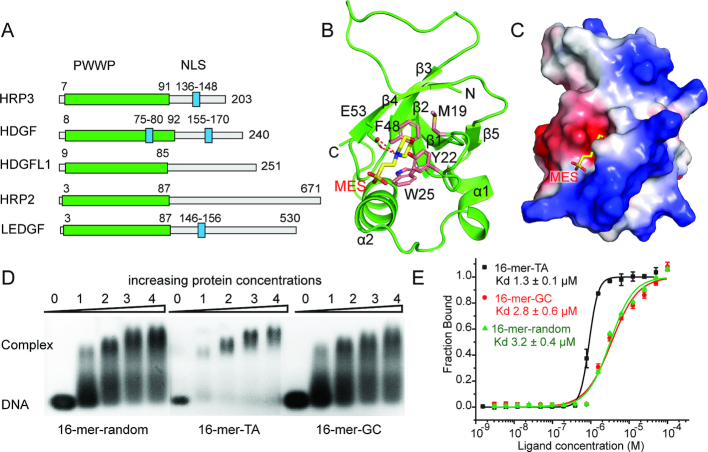
Apo-form structure of the HRP3 PWWP domain and its DNA binding analysis. (**A**) Domain architecture of the HRP family of proteins. (**B**) Apo-form structure of the HRP3 PWWP domain. The PWWP domain is coloured green. Residues forming the aromatic cage are coloured pink. The MES molecule is coloured yellow. (**C**) Electrostatic surface view of HRP3 PWWP. The positively charged surface is coloured blue. The negatively charged surface is coloured red. (**D**) EMSA analysis of the binding of HRP3 PWWP to various dsDNAs. The protein to DNA molar ratio is listed above the lanes. (**E**) MST-based measurements of the binding affinities of HRP3 PWWP to various dsDNAs. Dissociation constants (*K*d) are listed within the panel.

Hepatoma-derived growth factor (HDGF)-related proteins (HRPs) include HDGF, HRP1-3 and lens epithelium-derived growth factor (LEDGF), all of which are characterized by a conserved N-terminal PWWP domain (also known as the HATH domain) and a variable C-terminal region (Figure 1A) (1). As the founding member of this family of proteins, HDGF has been extensively studied (2). HDGF plays key roles in the early development of many tissues and is involved in multiple biological processes, such as transcriptional regulation (3,4), growth and differentiation (5), as well as mitogenic function (6). HDGF is highly expressed in hepatocellular carcinoma (HCC) cells (7). The elevated expression of HDGF is related to many types of cancer (2), which, irrespective of cancer type, correlates with a poor prognosis (7,8). Although HRP3 (HDGFL3) shares high sequence homology with HDGF, studies on its structure and function are limited. HRP3 plays an essential role in the development of neurons and the brain (9,10). It has also been found to be frequently upregulated in human HCC cells and is required for their anchorage-independent growth (11), demonstrating that its functions are not restricted to those of a mitogenic factor.

The PWWP domain is the only conserved structural unit in HRPs and has been extensively studied in several members of this family of proteins (12–14). The PWWP domain, which was first identified in the WHSC1 protein (5), is a structural motif of ∼100 amino acids and exists in more than 20 human proteins. The name of this domain is derived from a conserved Pro-Trp-Trp-Pro motif in WHSC1 (5), although only the third and fourth residues in the motif are conserved among various PWWP domains. Structural studies have revealed that the PWWP domain belongs to the Tudor domain ‘Royal Family’ (15). Similar to most members of the ‘Royal Family’, the PWWP domain also binds to methylated histones. Most PWWP domains prefer to bind to histone H3 tri- or di-methylated at Lys36 (H3K36me3/2) (12,16–20), which is important for the functions of HRPs during DNA repair (21–23) or transcriptional regulation (19,24–26). Some PWWP domains have been shown to bind H3K79me3 or H4K20me3/1 (12,27). A typical feature of most PWWP domains is that they bind to methylated histone substrates very weakly, with dissociation constants of ∼1–4 millimolar (19,28). However, the contribution of this weak interaction to their association with chromatin remains elusive. PWWP domains also bind to double-stranded DNA (dsDNA) without sequence preference (4,28–31). Therefore, the combined recognition of both methylated histones and dsDNAs implies that methylated nucleosomes are the preferred substrates for the PWWP domain, and this preference was verified by studies using designer nucleosomes as substrates (17,28,31,32). However, the detailed mechanisms by which the PWWP domain recognizes both DNA and histone substrates simultaneously and where PWWP domain-containing proteins are recruited *in vivo* are not clear.

In this study, we investigated the molecular mechanism of the PWWP domain of HRP3. We solved the high-resolution crystal structures of the HRP3 PWWP domain in apo-form and in complexed forms with/without bound methylated histone peptides in the presence of dsDNA. HRP3 PWWP binds to the minor groove of dsDNA through two loops and shows a preference for TA-rich sequences *in vitro*. HRP3 PWWP also recognizes the H3K36me3/2 modification through an aromatic cage. In addition, the HRP3 PWWP domain recognizes both the methylated histone peptide and dsDNA through two neighbouring surfaces, enabling us to generate a model illustrating the recruitment of PWWP to the nucleosomal substrate. *In vivo* analysis showed that HRP3 binds to genomic regions characterized by an accessible chromatin state. DNA binding plays a major role in HRP3 recruitment *in vivo*. Our work provides a molecular basis for understanding the mechanism of HRP3 recruitment to chromatin, shedding light on the study of other PWWP-containing proteins.

## MATERIALS AND METHODS

### Protein expression and purification

Full-length HRP3 and its truncations containing fragments 8–94, 1–99 and 1–110 were cloned into a modified pRSFDuet-1 vector bearing a 6хHis-SUMO-tag. Point mutations were generated using the QuikChange Site-Directed Mutagenesis Kit (Agilent). The target protein was expressed in cells of *Escherichia coli* strain Rosetta (DE3) at 37°C until the OD_600_ reached ∼1.0. The media was then cooled at 20°C for ∼1 h before 0.2 mM Isopropyl β-D-1-thiogalactopyranoside (IPTG) was added to induce expression overnight. Cells were harvested by centrifugation at 4500 *g* for 20 min at 4°C. Cell pellets were re-suspended in buffer containing 20 mM Tris at pH 7.0, 500 mM NaCl and 20 mM imidazole and then lysed by sonication. The cell lysate was centrifuged at 25 000 g for 1 h, and the supernatant was collected and loaded onto a nickel-charged HiTrap Chelating FF column (GE Healthcare). The His-SUMO-tagged target protein was eluted in buffer containing 20 mM Tris at pH 7.0, 500 mM NaCl and 500 mM imidazole and then cleaved by a His-tagged ULP1 protease. Both the His-SUMO tag and the ULP1 protease were removed by reloading the mixture onto a nickel-charged chelating column. The flow-through was pooled and further purified by a HiLoad 200 16/600 gel filtration column equilibrated with buffer containing 20 mM Tris, pH 7.0, 100 mM NaCl and 2 mM DL-Dithiothreitol (DTT). After gel filtration, the target protein was of high purity and ready for subsequent studies. Purified proteins were concentrated to ∼20 mg/ml and stored at −80°C.

### Crystallization and structure resolution

Crystallization was conducted using the sitting drop vapour diffusion method by mixing an equal volume of protein and well solution. Apo-form HRP3 PWWP (8–94) was crystallized with crystallization buffer containing 0.2 M ammonium sulfate, 0.1 M MES monohydrate, pH 6.5 and 30% w/v polyethylene glycol monomethyl ether 5000. The crystallization buffer containing 12% 2,3-butanediol was used as the cryoprotectant.

To obtain the binary complex of HRP3 PWWP(1–99) with bound 16-mer-random (5′-CAGGCTGGTCTTGAAC-3′), 16-mer-TA (5′-TATATATATATATATA-3′) or 10-mer-GC (5′-GCGCGCGCGC-3′) dsDNA, the protein and DNA were mixed at a molar ratio of 2:1.2 and incubated at room temperature for 1 h. The crystals of the binary complex were grown in a crystallization buffer containing 0.1 M Tris at pH 8.0, 0.1 M sodium malonate at pH 8.0 and 26% polyethylene glycol 1000. The crystallization buffer containing 12% 2,3-butanediol was used as the cryoprotectant.

The ternary complex of HRP3 PWWP(1–99)/16-mer-TA/H3_(33–40)_K36me3/2 was prepared by mixing the protein, DNA and histone peptide at a molar ratio of 2:1.2:6. The mixture was then incubated at room temperature for 1 h. Crystals were grown in a crystallization buffer containing 0.2 M NaCl, 0.1 M HEPES at pH 7.5 and 25% w/v polyethylene glycol 3350. The crystallization buffer containing 25% w/v polyvinylpyrrolidone K15 was used as the cryoprotectant.

Datasets for all crystals were collected at the beamlines of the Shanghai Synchrotron Radiation Facility (SSRF). The datasets were processed using HKL2000 (33), and the structures were solved by molecular replacement using the programme PHENIX (34) with the HDGF2 PWWP domain (PDB number: 3EAE) as the search model. The initial model was rebuilt in COOT (35) and further refined by PHENIX.

### EMSA

About 150 pM dsDNA was incubated with different concentrations of the target protein at room temperature for 20 min. The samples were then loaded onto a 1.2% agarose gel using 0.5× TAE as the running buffer. Electrophoresis was performed at 4°C for 20 min and the resulting gels were visualized by ethidium bromide staining.

To analyse the binding affinity of HRP3 PWWP to nucleosomal substrate, 1 pM native nucleosome or H3K_C_36me3-modified nucleosome was pre-mixed with PWWP in TCS buffer (20 mM Tris at pH 7.5, 2 mM EDTA and 2 mM DTT) at room temperature for 30 min. The mixtures were then loaded directly onto a 6% native polyacrylamide gel (60:1) in 0.2× TBE buffer. Electrophoresis was performed at 4°C for 1 h at a constant voltage of 150 V. The resulting gels were stained with ethidium bromide.

### ITC measurements

Isothermal titration calorimetry-based measurements were carried out at 20°C with a MicroCal iTC200 instrument. HRP3 PWWP (1–110) and various dsDNAs were dialysed overnight at 4°C in titration buffer containing 10 mM phosphate-buffered saline (PBS) at pH 7.0, 50 mM NaCl and 2 mM β-mercaptoethanol. Calorimetric titration data were fitted with the Origin software under the algorithm of one binding-site model.

### Mircoscale thermophoresis (MST) assay

All Mircoscale thermophoresis (MST) experiments were performed on a Monolith NT115 machine with 20% MST power and 100% LED power. All protein and DNA samples were dissolved in solution containing 50 mM KCl, 10 mM sodium cacodylate at pH 6.5 and 2 mM DTT. The MST samples were prepared by mixing HRP3(1-110) at concentrations ranging from 3 nM to 100 μM into a solution containing 200 nM 5′-FAM-labelled dsDNA. Experiments were performed using Monolith NT115 Standard Treated Capillaries, MO-K002. Dissociation constants were fitted with the MO Affinity Analysis software.

The DNA sequences used in this assay are as follows: 5′-FAM-16TA: 5′-TATATATATATATATA-3′; 5′-FAM-16GC: 5′-GCGCGCGCGCGCGCGC-3′; and 5′-FAM-16random: 5′-CAGGCTGGTCTTGAAC-3′.

### Nucleosome reconstitution

The *Xenopus laevis* core histones, H2A, H2B, H3 and H4, and 147-bp dsDNA with the ‘widom’ 601 sequence were prepared as previously described (36,37). H3K36 tri-methylation was introduced into histone H3 using the methyl-lysine analogue (MLA) method (38). The efficient incorporation of the MLA analogue was verified by mass spectrometry analysis.

### NMR titration experiments

All 2D ^1^H-^15^N HSQC NMR spectra were collected on the Bruker Avance III HD 600 MHz and 800 MHz spectrometers at 298 K. The NMR data were processed by NMRPipe (39) and analysed by KUJIRA (40) running with NMRview (41) softwares. Chemical shift perturbation experiments were carried out using ^15^N-labelled HRP3 PWWP (residues 1–110) dissolved in buffer containing 20 mM PBS at pH 6.9, 20 mM NaCl and 2 mM DTT. A series of 2D ^1^H-^15^N HSQC spectra were recorded at HRP3 PWWP:H3K36me3/2/1/0 (residues 29–41) molar ratios of 1:0, 1:0.5, 1:1.5, 1:4, 1:6, 1:10, 1:15 and 1:20, respectively. The combined chemical shift perturbation was calculated using the following equation: Δδ = [(Δδ^1^H)^2^ + (Δδ^15^N/5)^2^]^1/2^. Dissociation constants were calculated by fitting the data to a single-site ligand-binding model (SigmaPlot).

### Computational analysis of DNA geometry and electrostatic potential

DNA geometry was analysed with the 3DNA (42) and Curves+ (43) programs. Electrostatic potentials were calculated using the DelPhi program (44). The minor groove width was measured according to the method described (45). In detail, the distances between the *i*th (*i* indicates the number of the base from one chain) phosphate atom from one chain and the (*i* + 4)th atom from the paired chain were used to describe the minor groove width at the *i*th base. Electrostatic potentials were calculated at the midpoints between the above phosphate atom pairs used to calculate the minor groove widths.

For electrostatic calculations, Delphi version 8.1 was used with the following parameters. A scale of 4 grids/Å was used to draw a computational box at the centre of which the solute structures were placed. The box was designed with dimensions such that the solute would occupy only 70% of its total volume. A solvent probe of radius 1.4 Å was used to delineate a molecular surface separating the solute and the solvent phase. The solute region was assigned a dielectric of 2 and the exterior solvent region was assigned a dielectric of 80 with a physiological salt concentration of 0.145 M.

Before running Delphi to compute the electrostatic potential, the DNA nucleotides were protonated using DelPhiPKa (46) at the pH of 7.4. Partial charges and van Der Waal radii were derived from AMBER99SB force field.

The points at which the potentials were calculated were first mapped onto the nearest grid-point in the computational box. The potential at that grid-point in conjunction with that on the six of the neighbouring points along the *x, y* and *z* directions was used to compute the average value at the coordinate in question.

### Cell culture and cell line

HepG2 (ATCC HB-8065) and HEK293T human embryonic kidney cells (ATCC #CRL-11268) were cultured in Dulbecco’s modified Eagle’s medium supplemented with 10% FBS. Stable overexpression of wild-type or mutant HRP3 in HepG2 cells was performed using FUGW lentiviral constructs expressing wild-type or mutant HRP3. Lentiviral vector infections (47) and plasmid transfections (48) were performed as previously described. Overexpression efficiency was determined by western blot.

The lentiviral vector containing wild-type HRP3 was constructed by inserting the HRP3 AgeI/EcoRI fragment amplified from the Puc57 vector (Sangon Biotech) into the AgeI/EcoRI-digested FUGW vector (Addgene). HRP3 mutants were generated by two-step PCR mutagenesis. All constructs were verified by sequencing.

### Subcellular fractionation

Cellular fractionations were performed using a Subcellular Fractionation Kit (Thermo Scientific, cat. 78840) according to the manufacturer’s protocol. A total of 1 × 10^6^ cells were used for each cell line’s fractionation. Soluble nuclear contents and chromatin-bound nuclear contents were analysed by western blotting using anti-GFP (gta-20, Chromo-Tek) and anti-histone H3 (4499S, CST) antibodies.

### Immunofluorescence analysis

The cells were first fixed with 4% paraformaldehyde in PBS buffer for 30 min at room temperature, and then washed with PBS buffer twice. The cells were permeabilized with 0.5% Triton X-100 in PBS buffer for 10 min followed with PBS washing twice. Then, the cells were incubated with1 μg/ml 4′,6-diamidino-2-phenylindole (DAPI) (C0060, Solarbio) in PBS for 5 min. After washing with PBS buffer twice, the cells were ready for microscopic observation. 3D structural illumination microscopy was performed using N-SIM Super-resolution Microscope System (Nikon) with an objective lens CFI Plan (Apochromat Lambda 40 ×, 0.95 numerical aperture; Nikon).

### Chromatin immunoprecipitation

For chromatin immunoprecipitation, HepG2 cells were cross-linked with 1% formaldehyde at 37°C for 10 min and quenched with 0.125 M glycine for 5 min at room temperature. ChIP was performed using a ChIP assay kit (Millipore) according to the manufacturer’s protocol. Antibodies used for ChIP were GFP (gta-20, Chromo-Tek) and H3K36me3 (ab9050, abcam).

### Bioinformatics analysis

ChIP-Seq data were aligned to human genome hg19 using Bowtie 1.0 allowing one mismatch. Two biological replicates were performed and combined for the analysis. Analysis was performed using the Galaxy (49) and Cistrome (50) Platform and via custom R scripts for Bioconductor (51). Peak calling was performed using MACS2. Promoter definitions were downloaded from the UCSC browser (52). DNase I hypersensitivity and MNase-Seq data from ENCODE (GSM816662, GSM920557) (53) were used.

## RESULTS

### Structure of the apo-form HRP3 PWWP domain and its DNA-binding properties

We solved the crystal structure of the apo-form HRP3 PWWP (8–94) at an atomic resolution of 0.95 Å, which enabled us to build a model of the PWWP domain with high precision (Supplementary Table S1). The HRP3 PWWP domain has the characteristic fold of a PWWP domain, which contains a five-stranded β-barrel core followed by two α helices (Figure 1B). At the top of the β-barrel and sandwiched by the loops between β strands 1-2 and 3-4, an aromatic cage was formed. The aromatic cage, which is composed of three aromatic residues, Try22, Trp25 and Phe48, and a hydrophobic residue, Met19, constitutes the potential binding pocket for methylated histones (Figure 1B), as shown in several domains from the Tudor domain ‘Royal family’ (54,55). In the apo-form structure, an MES molecule from the crystallization buffer is positioned in the middle of the aromatic cage (Figure 1B). The hydrophobic head of the MES molecule is stabilized by the hydrophobic side chains of the aromatic pocket. In addition, two hydrogen bonds between Glu53 and the nitrogen atom of the MES molecule further strengthen the interaction. This binding mode provides implications for designing small molecule inhibitors targeting this aromatic cage. The PWWP domains have been shown to bind dsDNA (56). The electrostatic potential surface revealed that the surface next to the aromatic cage formed by helix 1 and the loop between β strands 1 and 2 may represent the DNA-binding surface, as it is highly basic (Figure 1C). EMSA verified that HRP3 PWWP binds to the various dsDNAs tested (Figure 1D), consistent with previous findings that the PWWP domain binds to DNA in a non-specific manner (57,58). The MST-based measurements showed that HRP3 PWWP bound to a randomly designed 16-bp dsDNA (16-mer-random) and a 16-bp GC-rich DNA (16-mer-GC) at comparable affinity, with dissociation constants of 3.2 and 2.8 μM, respectively (Figure 1E). However, it bound to a 16-bp TA-rich DNA (16-mer-TA) at 2–3-fold higher affinity, with a dissociation constant of 1.3 μM (Figure 1E). This TA-rich preference was further verified by ITC-based measurements (Supplementary Figure S1).

### The PWWP domain recognizes the minor groove of dsDNA

**Figure 2.**
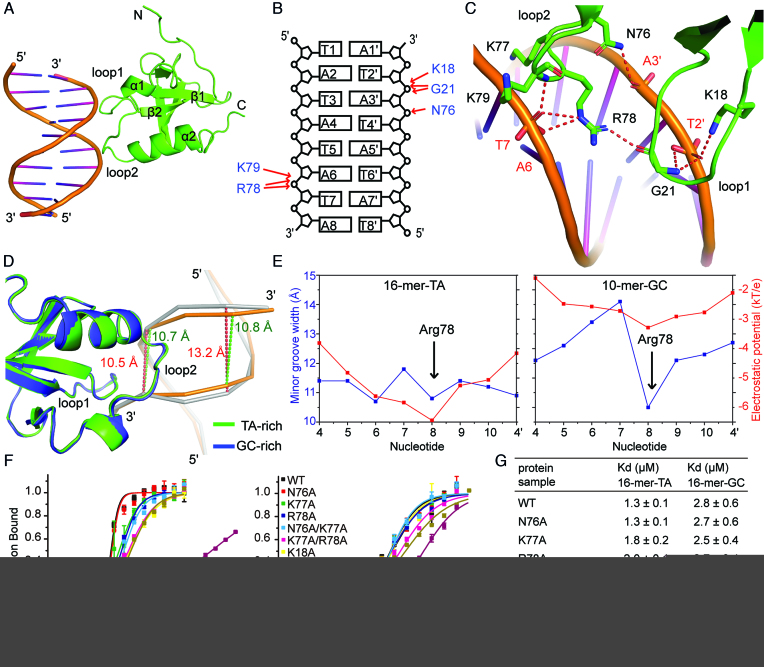
Structural details of the HRP3 PWWP and dsDNA complex and related mutational analysis. (**A**) Structure of the HRP3 PWWP and 16-mer-TA dsDNA complex. (**B**) Schematic representation of the interactions between HRP3 PWWP and the 16-mer-TA DNA. The DNA sequence is arbitrarily assigned. (**C**) Details of the interaction between HRP3 PWWP and the minor groove of the 16-mer-TA DNA. (**D**) Ribbon representation of the overlapped structures of the PWWP/TA-rich-DNA and PWWP/GC-rich-DNA complexes. In the TA-rich complex, PWWP is coloured green and the bound DNA is coloured orange. In the GC-rich complex, PWWP is coloured blue and the bound DNA is coloured grey. (**E**) Graphs comparing the minor groove widths (Å) and electrostatic potentials (*kT/e*) for 16-mer-TA and 10-mer-GC DNA molecules. (**F**) MST-based measurements of the dissociation constants of wild-type or mutant HRP3 PWWP for 16-mer-TA DNA (left panel) or 16-mer-GC DNA (right panel). (**G**) A table listing the dissociation constants measured in (F).

To understand the molecular mechanism of DNA recognition by HRP3 PWWP, we crystallized it with 16-mer-random DNA, 16-mer-TA DNA and 10-bp GC-rich DNA and solved their structures at resolutions of 2.0, 1.85 and 2.2 Å, respectively (Supplementary Table S1). In all of these structures, the DNA components in the crystals stacked one by one to form a 3D lattice, with the PWWP domain evenly positioned on the DNA network through its positively charged surface. The DNA bases could not be unambiguously identified, so the DNA sequences in both structures of the TA- and GC-rich DNA-containing complexes were randomly assigned from alternative choices. One PWWP domain spans 6 base pairs (bps). Unexpectedly, HRP3 PWWP binds to dsDNA mainly by recognizing the phosphate backbone of the minor groove but not by recognizing any specific base (Figure 2A and B). The loops between β strands 1-2 (designated as loop 1) and α helices 1-2 (designated as loop 2) are responsible for this recognition. Lys18 and Gly21 are the key residues on loop 1 that directly contact the backbone of one strand of the dsDNA molecule. The side chain of Lys18 and the main chain of Gly21 form a hydrogen bond with the phosphate backbone of T2′, respectively (Figure 2B and C). The main chain of Gly21 forms an additional hydrogen bond with the phosphoester linkage bonded oxygen atom connecting T2′ and A3′ (Figure 2C). Asn76, Arg78 and Lys79 are the residues on loop 2 that make direct contact with the backbone atoms from both strands. Asn76 forms a hydrogen bond with the phosphate group of A3′ (Figure 2C). The side chain of Arg78 and the main chain of Lys79 form a hydrogen bond with the phosphate backbone of T7, respectively (Figure 2C). In addition, the side chain of Arg78 forms an additional hydrogen bond with the phosphoester linkage bonded oxygen atom connecting A7 and A6 (Figure 2C). Arg78 also forms a hydrogen bond with the carbonyl oxygen of Gly21, which connects both loops into one structural unit during DNA recognition. In addition to the above-mentioned hydrogen bond interactions, several basic residues located in both loops, such as Lys77, which is positioned between the two DNA strands (Figure 2C), may also contribute to DNA binding through electrostatic interactions.

To understand the molecular basis of the TA-rich sequence preference by HRP3 PWWP, we superimposed the structures of the HRP3/TA-rich-DNA and HRP3/GC-rich-DNA complexes (Figure 2D). In both structures, the PWWP counterparts are well superimposed, with an R.M.S.D. of 0.33 Å over 98 equivalent backbone atoms. However, the bound DNAs showed clear differences. For the TA-rich-DNA containing complex, the width of the minor groove of the bound DNA showed almost no change between the PWWP-free region and the PWWP-binding region (the widths were 10.8 and 10.7 Å, respectively). However, for the GC-rich DNA-containing complex, the width of the minor groove of bound DNA was narrowed by ∼2.7 Å upon PWWP binding (changed from 13.2 to 10.5 Å). This result indicates that the width of the minor groove of the TA-rich DNA is more suitable for PWWP binding, whereas the GC-rich DNA has a wider minor groove that needs to be narrowed to accommodate efficiently HRP3 PWWP. This necessity may account for the reduced binding for GC-rich DNAs.

TA-rich sequences often form narrow minor grooves due to negative propeller twisting (59). Consistently, DNA geometry analysis by 3DNA (42) showed that the average propeller twist is −11.2° for 16-mer-TA and −7.3° for 10-mer-GC. In addition, the average helical twist is 37.1° for the 16-mer-TA DNA and 35.3° for the 10-mer-GC DNA. This means that it takes fewer TA-rich base pairs to form a helical turn, which may also facilitate the formation of a narrow minor groove. The shape of a DNA molecule will affect its electrostatic potentials (60). It has been shown that electrostatic focusing in the narrow grooves generates a negative electrostatic potential, which would facilitate the binding of positively charged arginines (61,62). Further analysis showed that Arg78 in HRP3 may play a role in the DNA-shape selectivity, as it not only forms direct hydrogen bonds with the phosphate backbone (Figure 2C), but is also positioned right in the middle of the narrowest region of the DNA minor groove (Figure 2E). We calculated the electrostatic potentials in the minor groove for both the TA-rich and GC-rich DNAs by the DelPhi program (44) and found that the widths of the minor groove correlated perfectly with the magnitudes of the negative electrostatic potential in both DNA structures (Figure 2E). For the 16-mer-TA DNA, the DNA minor groove widths had two minimum values. Arg78 bound at one of the width minima, where the electrostatic potential also reached a minimum (Figure 2E). For the 10-mer-GC DNA, Arg78 was located at the site of the minimum values of both the minor groove width and the electrostatic potential (Figure 2E). In addition, the difference in the average electrostatic potential between the calculated sites for both DNAs is 2.6 *kT/e*, which could explain the higher binding affinity for the TA-rich DNA molecules. To test whether PWWP binding would induce a global bending of the target DNA, we analysed the curvature of both TA- and GC-rich DNAs in their complexes with the Curve+ software (43). We found that both DNAs showed a very small bending angle towards the minor groove (2.9° per 6 bps for 16-mer-TA and 3.4° per 6 bps for 10-mer-GC), indicating that PWWP binding did not induce noticeable bending of the target DNA.

To verify the residues important for DNA recognition, we made single or combined mutations of the residues on both loops 1 and 2 of PWWP and examined their binding to dsDNA through MST-based analysis. We found that residues Lys77 and Arg78 from loop 2 and residue Lys18 from loop 1 all played a role in the recognition of the target DNA, as a single mutation of these residues resulted in a 1.4- to 2.2-fold loss of binding for the 16-mer-TA DNA (Figure 2F–G). As a control, the N76A mutation did not have an impact on the binding affinity. Unexpectedly, single mutations of the above residues did not change the binding to the 16-mer-GC DNA (Figure 2F and G). This indicates that key residues from both loops, such as Arg78 in loop 2 and Lys18 in loop 1, play a role in the selection for TA-rich sequences. We also found that both loops functioned in coordination for DNA recognition. Single or combined mutations on either loop alone resulted in a <2.5-fold decrease in binding affinity. However, the mutant K18A/N76A/R78A bearing key residue mutations from both loops resulted in a 58-fold and a 5.7-fold loss of binding with 16-mer-TA and 16-mer-GC substrates, respectively (Figure 2F and G).

### The HRP3 PWWP domain recognizes H3K36me3-containing histone tails

**Figure 3.**
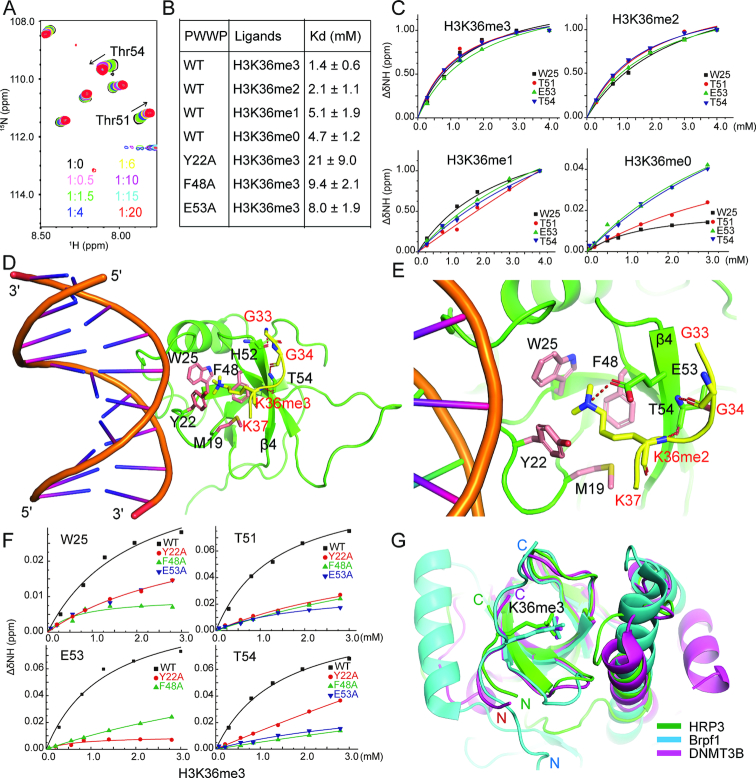
HRP3 PWWP binds to the H3K36me3/2-containing histone peptide. (**A**) A panel of overlapping HSQC spectra of the HRP3 PWWP domain with various concentrations of the H3K36me3 peptide. (**B**) A table listing the NMR-based measurements of the dissociation constants for the binding between the wild-type or mutant forms of HRP3 PWWP and various H3K36-containing peptides with/without modifications. (**C**) NMR-based measurements of different dissociation constants of HRP3 PWWP interacting with H3K36me3/2/1/0-containing histone peptides. (**D**) Structural details of the ternary complex of HRP3 PWWP/dsDNA/H3K36me3-peptide. The histone peptide is coloured yellow. (**E**) Structural details of the ternary complex of HRP3 PWWP/dsDNA/H3K36me2-peptide. (**F**) NMR-based measurements of the dissociation constants for the binding of the different HRP3 PWWP mutants with the H3K36me3 peptides. (**G**) Overlapped structures of the PWWP domain from HRP3 (in green), Brpf1 (in cyan) and DNMT3B (in magenta) with bound H3K36me3 peptides.

As histone H3 tri- or di-methylated at lysine 36 (H3K36me3/2) is a preferred substrate for most PWWP-containing proteins (56), we tested the interaction between HRP3 PWWP and an H3 histone peptide methylated at Lys36 using the NMR titration method. As the concentration of the added H3K36me3 peptide increased, the chemical shift in several residues of PWWP also changed, indicating a sequence-specific interaction (Figure 3A and Supplementary Figure S2). HRP3 PWWP showed a preference for the H3K36me3/2-containing substrate over the H3K36me1/0-containing substrates (Figure 3B and C). The dissociation constants for H3K36me3- and H3K36me2-containing peptides were 1.3 and 2.1 mM, respectively. To reveal the molecular basis of the H3K36me3/2-specific recognition by the PWWP domain, we crystallized the complex of HRP3 PWWP with a bound H3K36me3- or H3K36me2-containing peptide in the presence of a 10-bp dsDNA and solved their structures at resolutions of 2.4 and 2.1 Å, respectively (Supplementary Table S1). In both the PWWP-DNA-H3K36me3/2 ternary complexes, the histone peptide extends parallel to strand β4 (Figure 3D and E). In the PWWP-DNA-H3K36me3 ternary complex, the main chain atoms from Gly33, Gly34 and Lys36me3 of the H3 histone peptide form three hydrogen bonds with the main chain atoms from His52 and Thr54 of PWWP (Figure 3D), which stabilize the interaction. In the PWWP-DNA-H3K36me2 ternary complex, two hydrogen bonds were observed between the main chain atoms from Gly34 and Lys36me2 of H3 and the main chain atoms of Thr54 located on strand β4 of PWWP (Figure 3E). The side chains of both the tri- and di-methylated Lys36 are positioned in the aromatic cage composed of the residues Met19, Tyr22, Trp25 and Phe48 (Figure 3D and E), as identified in the apo-form structure, where they are stabilized by cation–π interactions (63). One major difference between both complexes is that the dimethylated side chain of Lys36 forms a direct salt bridge with Glu53 of PWWP in the H3K36me2-containing complex (Figure 3E). Consistent with the above structural analysis, single mutations in the aromatic cage residues, Y22A and F48A, resulted in a 15-fold and a 6.7-fold loss of binding affinity for the H3K36me3 peptide, respectively (Figure 3B and F). The E53A mutation also decreased the binding of the H3K36me3 peptide by 5.7-fold (Figure 3B and F).

Several PWWP domains have been shown to bind H3K36me3/2-containing histone peptides (12,16,17,19,20). Superimposition of the PWWP domain of HRP3 with those from Brpf1 (19) and DNMT3B (16) revealed that despite the large divergence in their C-terminal α-helix-containing regions as discussed previously (12,56), their central β strand barrels were well superimposed (Figure 3G). Consistently, the bound H3K36me3-containing peptides displayed a similar binding mode in all three proteins (Figure 3G). This further verified that HRP3 PWWP recognizes H3K36me3/2.

### Model of nucleosome-based recognition by the HRP3 PWWP domain

**Figure 4.**
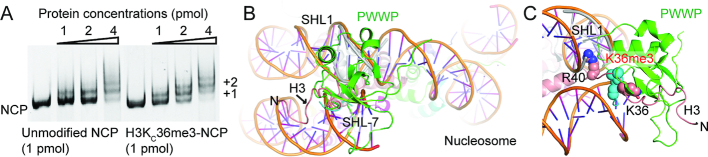
Model of HRP3 PWWP recruitment on the H3K36me3-containing nucleosome. (**A**) EMSA analysis of HRP3 PWWP with the unmodified nucleosome or with the H3K_C_36me3-modified nucleosome. +1 or +2 indicates one or two HRP3 PWWP binding to a single nucleosome. (**B**) A side overview of the HRP3-nucleosome model. The PWWP domain is coloured green. PWWP-bound DNA coloured in grey is docked into a region of the SHL1 minor groove. Histone H3 N-terminal tails are coloured salmon. (**C**) A zoomed view of the HRP3-nucleosome model. The N-terminal H3 tail, HRP3 PWWP and its bound DNA are coloured the same as in (B). HRP3-bound histone peptide is coloured cyan. The residues H3K36 and H3R40 in the nucleosome and H3K36me3 in the HRP3 complex are shown as spheres.

While the PWWP domain can bind both dsDNAs and modified histones, several PWWP domain-containing proteins have been shown to prefer to bind to nucleosome-based substrates (17,28,31,32). To test whether HRP3 PWWP also prefers nucleosomal substrates, we used unmodified nucleosomes reconstituted with a recombinant histone octamer wrapped with a 147-bp dsDNA and nucleosomes bearing H3K36me3 modifications on both H3 tails. We then compared their binding affinity for HRP3 PWWP using EMSAs. To introduce the H3K36me3 modification into the nucleosome, we used site-specific incorporation of the trimethylated lysine analogue into the recombinant H3 (38) (designated as H3K_C_36me3). The efficient incorporation of the trimethylated lysine analogue was verified by the mass spectra (Supplementary Figure S3). Nucleosomes bearing the H3K_C_36me3 analogues retain the functions of their natural counterparts (38). According to EMSA, the H3K_C_36me3 nucleosomes showed higher binding affinity than their unmodified counterparts (Figure 4A), suggesting that HRP3 PWWP also recognizes both dsDNA and the H3K36me3 modification on a nucleosomal substrate. Histone H3 segment 39–42 in the nucleosome is sandwiched between the juxtaposed minor grooves of superhelix locations SHL-7 and SHL1 (superhelix location was defined as the number of the DNA double helix relative to the central base pair at the pseudo-2-fold axis of the nucleosome particle) (64). Based on the histone peptide orientation in the HRP3 PWWP/DNA/histone complex, the minor groove of SHL1 is the only choice suitable for HRP3 PWWP recruitment. As the nucleosomal DNA showed various bending angles (64), we selected a region of the minor groove of SHL1 where the curvature and the minor groove width were suitable for HRP3 PWWP docking and generated a model to illustrate the recruitment of HRP3 PWWP to the nucleosome (Figure 4B and C). In this model, HRP3 PWWP is positioned near the linker DNA region between two layers of dsDNA, where it contacts the minor groove of SHL1 on the inner layer of dsDNA through one surface and touches the H3 tail extruded in-between two layers of the nucleosomal DNA through an adjacent surface (Figure 4B and C). By coincidence, the HRP3 docking site is very close to the Arg40 of histone H3, which is inserted into the minor groove of SHL1 in the nucleosomal DNA where the width is relatively narrow (Figure 4C). To accommodate HRP3 PWWP binding, the H3K36-containing region of the histone in the nucleosome would change its conformation a little bit to fit its binding site on the PWWP domain. Both the bound DNA and the histone peptide can be suitably docked onto their nucleosomal counterparts. Therefore, this model can reasonably explain the nucleosome-based recognition by the PWWP domain of HRP3.

### Both histone- and DNA-mediated recognition are important for HRP3 recruitment *in vivo*

**Figure 5.**
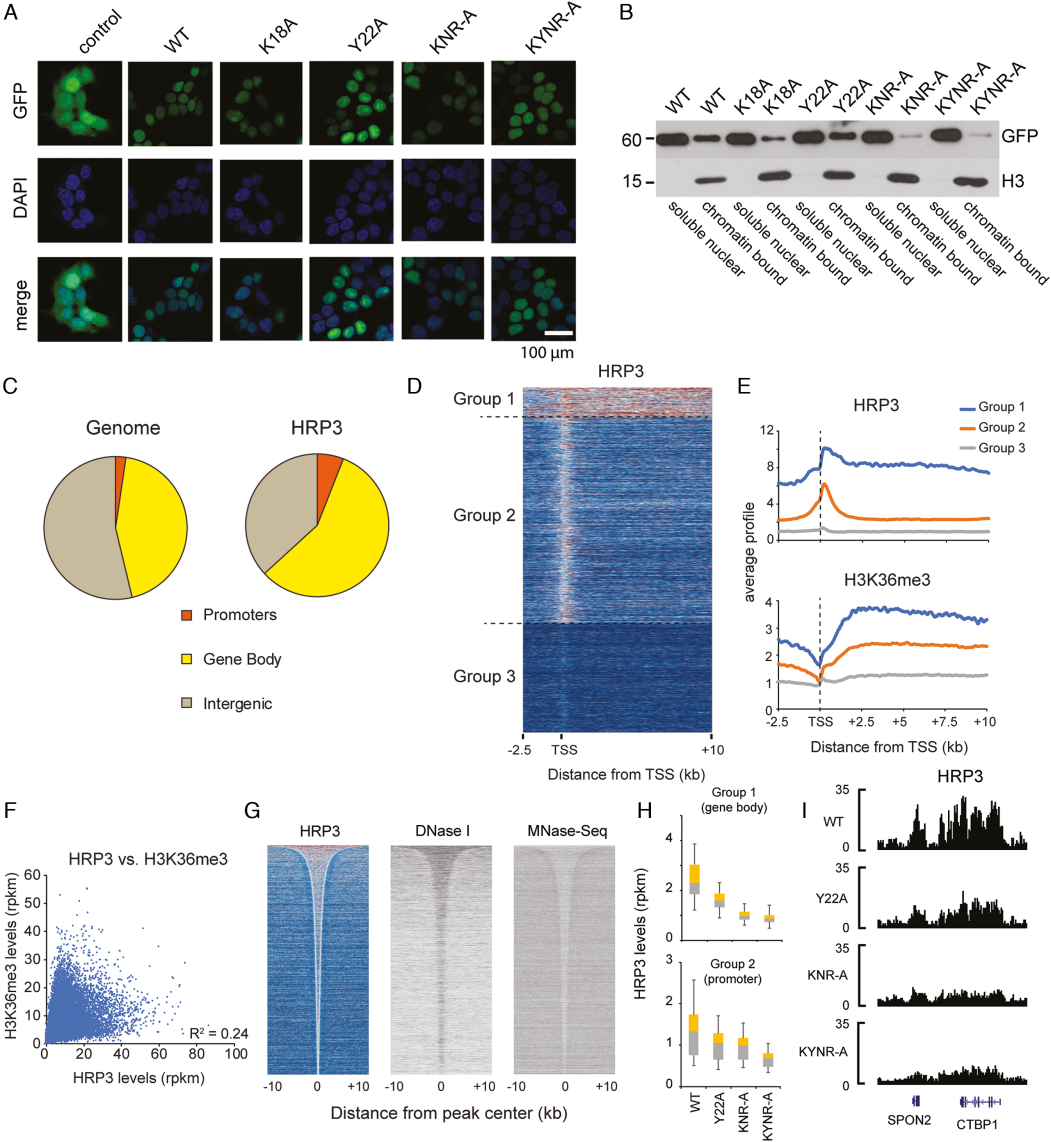
HRP3 binds to accessible chromatin. (**A**) Fluorescence microscopy of GFP-tagged HRP3 proteins in HepG2 cells, in comparison to GFP control. Scale bar, 100 μm. (**B**) Distribution of various ectopically expressed GFP-HRP3 variants in nucleoplasmic or chromatin fractions of HepG2 cells. (**C**) Genome-wide HRP3 distribution compared to the genome. (**D**) Three gene groups were identified based on HRP3 levels. Group I genes are characterized by very strong binding up- and downstream of the transcription start site. Group II genes have HRP3 mainly bound at the promoter region. Group III genes are not bound by HRP3. (**E**) Profiles of HRP3 and H3K36me3 in the three gene groups. (**F**) Relationship between HRP3 and H3K36me3 at gene bodies. (**G**) Heat maps showing all significant HRP3 peaks (*n* = 79 555), sorted by size, and the respective DNase I hypersensitivity and nucleosome density. (**H**) HRP3 levels of the wild-type and mutant HRP3 (KNR-A = K18A/N76A/R78A, KYNR-A = K18A/Y22A/N76A/R78A) at gene bodies of group I genes and promoters of group II genes. (**I**) Example at the *CTBP1* gene.

Next, we wanted to elucidate whether HRP3 plays a role in chromatin and gene regulation. We selected HepG2 cells as the model, given that HRP3 has been described to play a role in these cells (65). To address the chromatin-binding capacity of HRP3 *in vivo*, we ectopically expressed wild-type or several DNA-binding, histone-binding, or both DNA and histone binding mutant versions of human HRP3 as GFP fusion proteins in HepG2 cells. Through fluorescence microscopy, we found that HRP3 is enriched in the nucleus (Figure 5A), consistent with a nuclear function. Upon cellular fractionation experiments, we observed that mutation of the DNA binding amino acids (K18A, K18A/N76A/R78A, short as KNR-A and K18A/Y22A/N76A/R78A, short as KYNR-A), but not the H3K36me3 binding amino acids (Y22A) alone, dramatically reduced the chromatin binding of HRP3 (Figure 5B). To address this in more detail, we performed ChIP-Seq to check the chromatin recruitment of HRP3 using a GFP antibody. Upon initial investigation, we found that the wild-type HRP3 was enriched at promoters and gene bodies (Figure 5C and D). Many HRP3-binding sites reflect long stretches over several kilobases. Although the HRP3 binding pattern appears to correlate with H3K36me3 levels (Figure 5E), there are many instances where HRP3 but not H3K36me3 is present and vice versa (Figure 5F), suggesting that HRP3 binding may not or only partially depend on H3K36me3. Consistent with a rather unspecific binding of HRP3 to the DNA minor groove, we could not detect the enrichment of a specific DNA-binding motif. Thus, we speculated that other features may be more relevant for the chromatin binding of HRP3. Upon investigation of the relationship between HRP3 binding and chromatin accessibility, we found that the regions enriched for HRP3 have increased DNase I hypersensitivity and reduced nucleosome density (Figure 5G), implying that HRP3 prefers to bind to genomic locations with a loose nucleosome structure.

In line with our *in vitro* and *in vivo* studies (Figure 5B), DNA-binding mutations (both KNR-A and KYNR-A) lead to a strong reduction in chromatin binding (Figure 5H and I). In contrast, the mutation of the H3K36me3 binding pocket (Y22A) has weaker consequences (Figure 5I). These results suggest that the DNA-binding capacity of HRP3 is most critical for its chromatin association *in vivo*, although the H3K36me3 binding function may also contribute to chromatin binding. Collectively, our data suggest that HRP3 binds to accessible chromatin in a DNA binding-dependent manner.

### DISCUSSION

Although the PWWP domain is a small chromatin-associated module, it is very unique, as it can recognize both DNA and histone substrates simultaneously. Previous studies on the histone substrates of PWWP have determined that methylated histone substrates, especially histone H3 tri- or di-methylated at Lys36, are preferred substrates for most PWWP domains. In contrast, its DNA-binding properties remain elusive. Previous studies showed that the PWWP domain was able to bind dsDNAs non-specifically, with dissociation constants in the low nanomolar to high micromolar range (56). Several groups mapped the DNA-binding surface through NMR-based chemical shift perturbation experiments and identified similar DNA-binding surfaces to those shown in this study (56). However, due to a lack of structural information, they were not able to identify the key residues responsible for the recognition. Consequently, their models of nucleosomal recruitment were not accurate enough, as in all those models, the PWWP domains were docked onto the major groove of the nucleosomal DNA or a standard dsDNA (29,31,32).The complexed structure of HDGF PWWP with bound DNA, for the first time, revealed that the HDGF PWWP domain recognized the minor groove of a 10-bp dsDNA derived from the *SMYD1* promoter (58). Structural comparison showed that the HRP3 and HDGF PWWP domains displayed similar DNA-binding modes; that is, both proteins bind DNA by recognizing the phosphate backbone of the minor groove (Supplementary Figure S4). However, there are quite a few differences between both complexes. For example, HDGF PWWP formed a domain-swapped dimer to recognize the DNA substrate, but it is not clear whether such a dimer is required for the DNA recognition of other PWWP domains. In addition, it is not clear whether HDGF PWWP has a preferred sequence or structure. In this work, we solved the high-resolution crystal structures of HRP3 PWWP with various dsDNAs and clearly verified that the monomer HRP3 PWWP can efficiently recognize the minor groove of dsDNA. HRP3 PWWP contacts only the phosphate backbone of the minor groove of its bound dsDNA, explaining the non-sequence specific DNA-binding property of PWWP domains identified previously. Furthermore, we found that HRP3 PWWP is selective for DNA structure. HRP3 PWWP prefers to bind DNA sequences bearing a narrow minor groove. Consecutive ApA, TpT or ApT base pair steps lead to a narrow minor groove due to negative propeller twisting (59). The 16-mer-TA sequence used in this study has consecutive ApT steps, thus forming a much narrower minor groove than that of the 10-mer-GC DNA and showing a higher binding affinity to the HRP3 PWWP. The selectivity for a narrow minor groove width has been shown for other transcription regulators (61,62). In the complex structure of the Hox homeodomain Src with its bound DNA substrate, the minor groove selectivity is mediated by an arginine and a histidine, both of which are located at the narrower regions of the bound DNA minor groove (61). In HRP3 PWWP, Arg78 on loop 2 is also located at the narrowest site of its bound DNA, where a local electrostatic potential minimum is created due to the phenomenon of electrostatic focusing (60,66), which may explain the minor groove selectivity by this residue. Another important finding is that the minor groove recognition is mediated by the coordinating effects of both loops, as mutations on key residues from both loops (Lys18 from loop 1, Arg78 from loop 2), but not from either individual loop, resulted in a dramatic loss of binding affinity (Figure 2F). Sequence alignments of several subfamilies of PWWP domains from humans showed that for the other PWWP domains, the residue corresponding to Lys18 in loop 1 of HRP3 is conserved, while the residue corresponding to Arg78 in loop 2 of HRP3 is not conserved (Supplementary Figure S5). Despite the low sequence conservation, most of loop 2 motifs of those PWWP domains are very basic, and contain several basic residues. This indicates that most, if not all, of the other PWWP domains would have the potential to recognize the DNA minor groove through similar mechanisms (Supplementary Figure S5).

The DNA recognition mode by HRP3 PWWP is special and unique compared with several other well-known DNA-binding domains that exclusively interact with DNA through minor groove contacts. First, most minor-groove specific DNA-binding proteins prefer to bind DNA with some degree of sequence specificity (67), while HRP3 PWWP has selectivity only on DNA shape (a narrow minor groove) but not a specific sequence, as discussed above. Second, most minor groove-binding proteins induce to a dramatic widening and bending of the minor groove upon DNA recognition, as shown by TBP and HMG-box domain-containing proteins (67). In strong contrast, HRP3 PWWP binding caused little conformational change to its target DNAs. HRP3 PWWP either maintains the shape of the DNA minor groove if it is narrow enough (for TA-rich sequences) or induces a local dip of the minor groove (for GC-rich sequences). In addition, HRP3 PWWP binding caused neglectable bending of the target DNA. Taken together, the PWWP domain can be considered a new family of minor groove-specific DNA-binding domains, which extends the repertoire of minor groove-specific binding proteins.

HDGF-related proteins are closely related to human cancers. Large-scale sequencing data revealed quite a few somatic mutations of HDGF-related proteins in human cancer tissues. R78W mutation in HRP3 has been found in colorectal carcinoma (68). The corresponding residue in HDGF, Arg79 was also found mutated to a threonine in gastric cancer patients (69). Similarly, K19N mutation in HDGF (Lys19 in HDGF corresponds to Lys18 in HRP3) was found in squamous cell carcinoma (70). These mutations would disrupt the association of HRP3 or HDGF with the chromatin, which may lead to dysfunctions of both proteins. Further work is needed to elucidate the biological impact of HRP3 recruitment on chromatin and its relationship with human pathogenesis.

## DATA AVAILABILITY

Atomic coordinates and structure factors for the apo-form HRP3, two binary complexes of HRP3 with bound DNA and two ternary complexes of HRP3 with bound DNA and histone peptides were deposited in the protein data bank with the accession codes of 6IIP, 6IIQ, 6IIR, 6IIS and 6IIT, respectively. The ChIP-seq datasets are available at the GEO repository under accession number GSE120492.

## Supplementary Material

gkz294_Supplemental_File
