# Supplementary material for: The HRP3 PWWP domain recognizes the minor groove of double-stranded DNA and recruits HRP3 to chromatin
Source: Nucleic Acids Res. 2019 Apr 24;47(10):5436–48. doi: 10.1093/nar/gkz294 (PMC6547440; doi:10.1093/nar/gkz294)
Supplement: gkz294_Supplemental_File [file gkz294_supplemental_file.pdf]

Supplementary Data for

The HRP3 PWWP domain recognizes the minor groove of double-stranded DNA and recruits HRP3 to chromatin

Wei Tian, Peiqiang Yan, Ning Xu, Arghya Chakravorty, Robert Liefke*, Qiaoran Xi* and Zhanxin Wang*

*Email: robert.liefke@imt.uni-marburg.de; xiqiaoran@tsinghua.edu.cn; wangz@bnu.edu.cn;

This PDF file includes:

Supplementary Figures S1 to S5
Supplementary Table S1

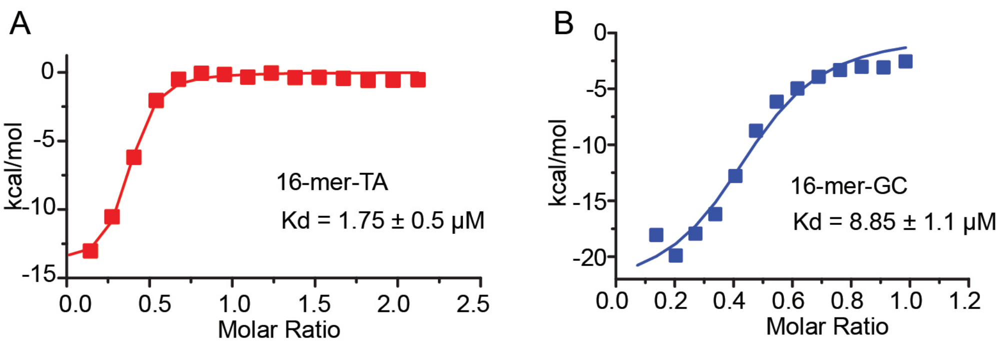

Supplementary Figure S1. ITC-based measurements of the binding affinities of HRP3 PWWP and various DNAs. (A, B) ITC-based binding curves of HRP3 PWWP titrated with the 16-mer-TA DNA (A) or the 16-mer-GC DNA (B).

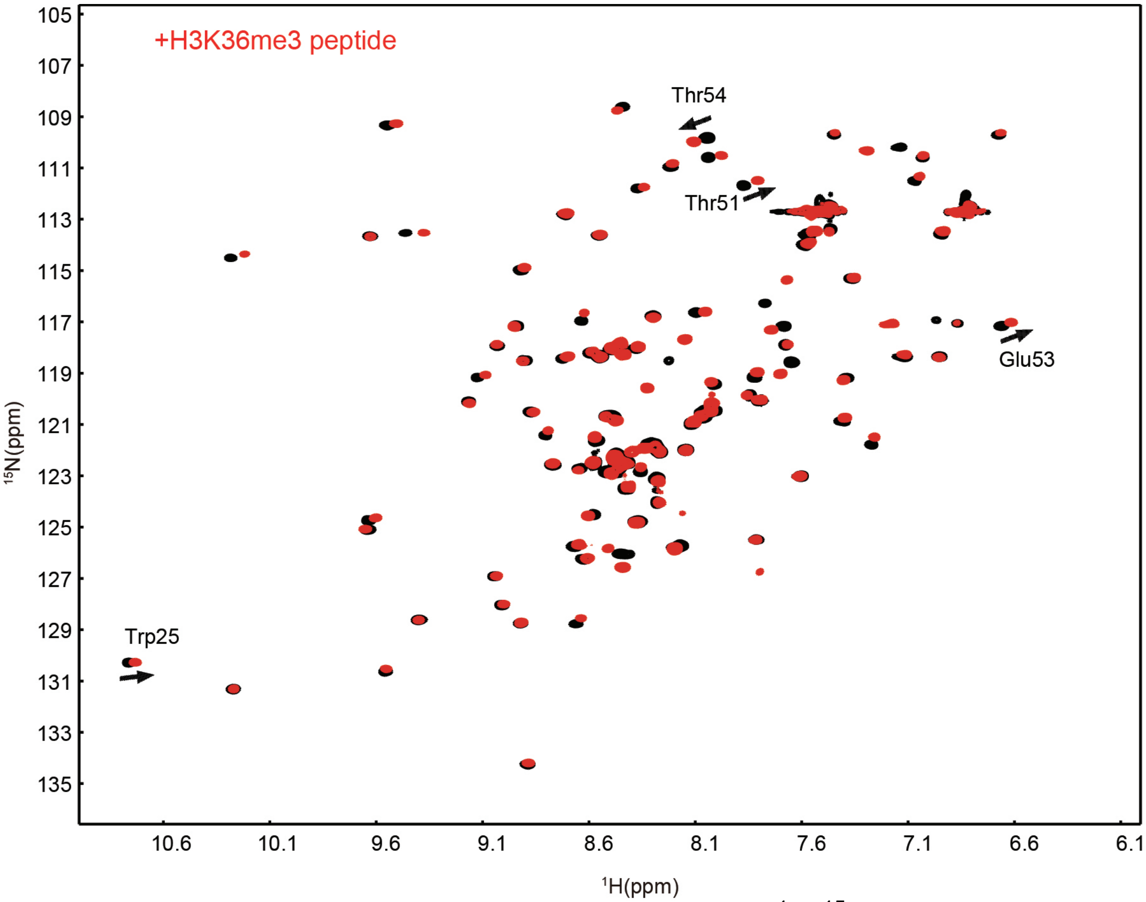

Supplementary Figure S2. Changes induced in the H^1-N^{15} HSQC NMR spectra of HRP3 PWWP upon binding of 4 mM H3K36me3 peptide. Superposition of the H^1-N^{15} HSQC NMR spectra of HRP3 PWWP in the absence (black) and presence (red) of 4 mM H3K36me3 peptide.

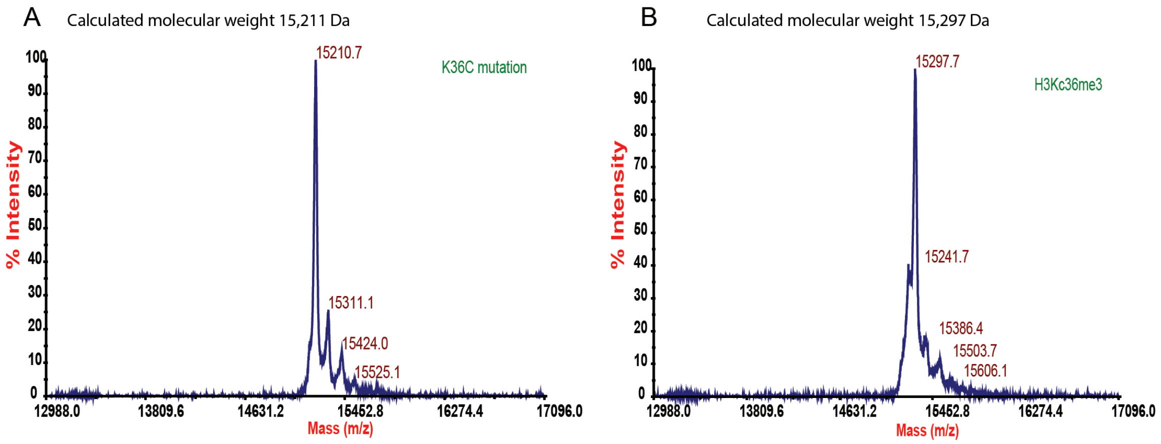

Supplementary Figure S3. Mass spectra to verify the incorporation of the trimethylation analogue of H3K36me3. (A, B) Mass spectra of H3 bearing the K36C mutation (A) and the trimethylation analogue of H3Kc36me3 (B). Calculated molecular weights of both peptides are indicated above the panels.

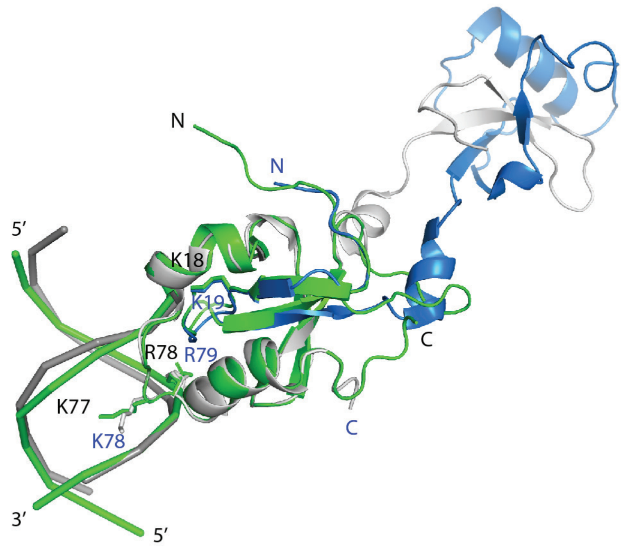

Supplementary Figure S4. Comparison of the DNA-bound structures of HRP3 PWWP and HDGF PWWP (PDB ID: 5XSK). HRP3 PWWP domain is coloured green. Swapped HDGF PWWP domains are coloured grey and blue, respectively. Key residues for the DNA recognition in HRP3 and HDGF are labelled in black and blue, respectively.

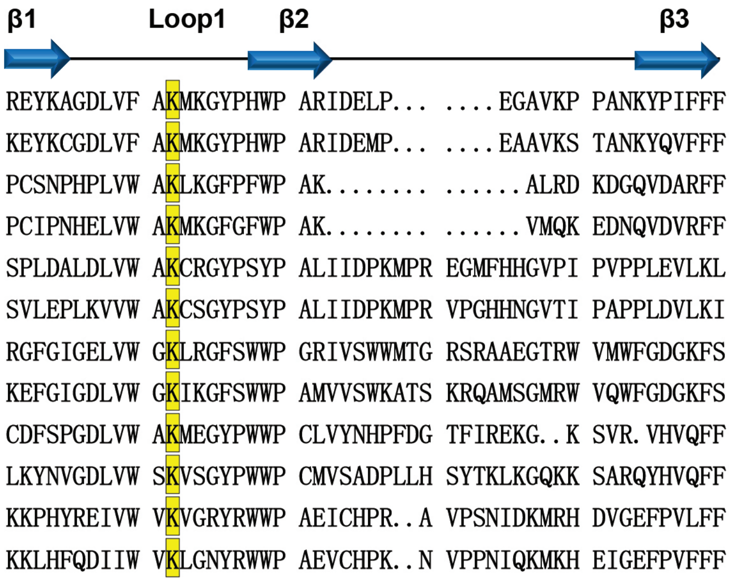


```
HRP3          7 REYKAGDLVF AKMKGYPHWP ARIDELP... ....EGAVKP PANKYPIFFF 495
HDGF          8 KEYKCGDLVF AKMKGYPHWP ARIDEMP... ....EAAVKS TANKYQVFFF 50
ZMYND8      268 PCSNPHPLVW AKLKGFPFWP AK........ ......ALRD KDGQVDARFF 308
ZMYND11     276 PCIPNHELVW AKMKGFGFWP AK........ ......VMQK EDNQVDVRFF 311
BRPF1      1081 SPLDALDLVW AKCRGYPSYP ALIIDPKMPR EGMFHHGVPI PVPPLEVLKL 1130
BRPF2       925 SVLEPLKVVW AKCSGYPSYP ALIIDPKMPR VPGHHNGVTI PAPPLDVLKI 974
DNMT3A      288 RGFGIGELVW GKLRGFSWWP GRIVSWWMTG RSRAAEGTRW VMWFGDGKFS 337
DNMT3B      221 KEFGIGDLVW GKIKGFSWWP AMVVSWKATS KRQAMSGMRW VQWFGDGKFS 270
MSH6         88 CDFSPGDLVW AKMEGYPWWP CLVYNHPFDG TFIREKG..K SVR.VHVQFF 134
NSD2_N      218 LKYNVGDLVW SKVSGYPWWP CMVSADPLLH SYTKLKGQKK SARQYHVQFF 267
NSD1_C     1752 KKPHYREIVW VKVGRYRWWP AEICHPR..A VPSNIDKMRH DVGEFPVLFF 1799
NSD2_C      876 KKLHFQDIIW VKLGNYRWWP AEVCHPK..N VPPNIQKMKH EIGEFPVFFF 923
```

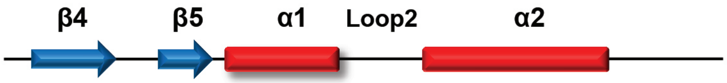


```
HRP3         50 GTHETAFLGP KDLFPYKEYK DKFGKSNKRK GFNEGLWEIE NNPGVKFTGY 99
HDGF         51 GTHETAFLGP KDLFPYEESK EKFGKPNKRK GFSEGLWEIE NNPTVKASGY 100
ZMYND8      309 GQH.DRAWVP INNCYLMSKE IPFSVKKTKS IFNSAMQEME VYVENIRRKF 357
ZMYND11     312 GHHHQRAWIP SENIQDITVN IHRLHVKRSM GWKKACDELE LHQRFLREGR 361
BRPF1      1131 GEQMTQEARE HLYLVLFFDN KRTWQWLPRT KLVPLGVNQD LDKEKMLEGR 1180
BRPF2       975 GEHMQTKSDE KLFLVLFFDN KRSWQWLPKS KMVPLGIDET IDKLKMMEGR 1024
DNMT3A      338 VVCVEKLMPL SSFCSAFHQA TYNKQPMYRK AIYEVLQVAS SRAGKLFPVC 387
DNMT3B      271 EVSADKLVAL GLFSQHFNLA TFNKLVSYRK AMYHALEKAR VRAGKTFPSS 320
MSH6        135 DDSPTRGWVS KRLLKPYTGS KSKE..AQKG GHFYSAKPEI LRAMQRADEA 182
NSD2_N      268 GDAPERAWIF EKSLVAFEGE GQFEKLCQES AKQAPTKAEK IKLLKPISGK 317
NSD1_C     1800 G.SNDYLWTH QARVFPYMEG DVSSKDKMGK GVDGTYKKAL QEAAARFEEL 1848
NSD2_C      924 G.SKDYYWTH QARVFPYMEG DRGSRYQGVR GIGRVFKNAL QEAEARFREI 972
```

Supplementary Figure S5. Sequence alignments of several PWWP domains. One or two human PWWP domains from each subfamily of PWWP-containing proteins are listed. Secondary structural elements from HRP3 PWWP are shown above the sequences. The conserved K18 in loop 1 of HRP3 PWWP and its counterparts in other PWWP domains are highlighted. R78 in loop 2 of HRP3 PWWP and its corresponding or neighboring basic residues in the other PWWP domains are color coded.

Supplementary Table S1. Data collection and refinement statistics

Crystal	Free PWWP(8-94)	PWWP(1-99) with 16-mer-TA	PWWP(1-99) with 10-mer-GC	PWWP(1-99) with DNA and H3(33-40)K36me3	PWWP(1-99) with DNA and H3(33-40)K36me2
PDB entry	6IIP	6IIQ	6IIR	6IIS	6IIT
Beam line	SSRF-BL19U1	SSRF-BL19U1	SSRF-BL19U1	SSRF-BL19U1	SSRF-BL17U1
Wavelength	0.91531	0.97853	0.97853	0.97853	0.97945
Space group	$P2_12_12_1$	$C2_1$	$P3_1$	$P3_1$	$P3_1$
Unit cell					
a, b, c (Å)	38.5, 39.9, 97.4	57.4, 33.6, 201.2	33.6, 33.6, 197.9	33.5, 33.5, 198.8	33.6, 33.6, 200.1
α, β, γ (^{o})	90.0, 90.0, 90.0	90.0, 90.0, 90.0	90.0, 90.0, 120.0	90.0, 90.0, 120.0	90.0, 90.0, 120.0
Resolution (Å)	50.0-0.95 (0.97-0.95)[a]	30.0-1.85 (1.89-1.85)	50.0-2.20 (2.24-2.20)	50.0-2.35 (2.39-2.35)	50.0-2.10 (2.14-2.10)
R_{sym}	0.072 (0.455)	0.084 (0.611)	0.111 (0.975)	0.087 (0.533)	0.135 (1.17)
I/σ (I)	52.6 (5.3)	22.3 (2.0)	35.9 (2.1)	16.0 (2.0)	36.5 (4.1)
Completeness (%)	97.4 (86.0)	94.3 (96.4)	99.9 (100)	91.6 (84.2)	100.0 (100.0)
Redundancy	11.9 (11.2)	4.2 (4.3)	7.5 (7.0)	3.6 (3.9)	7.2 (6.9)
Number of unique reflections	93005	31501	12832	9478	14978
R_{work}/R_{free} (%)	19.7/20.5	20.6/25.3	20.2/25.8	18.6/26.3	19.1/26.4
Number of non-H atoms					
Protein	1432	2432	1624	1632	1638
Peptide	/	/	/	72	70
DNA	/	615	410	410	410
Water	294	186	5	18	52
Average B factors (Å^2)					
Protein	10.4	36.5	79.3	47.4	43.5
Peptide	/	/	/	66.6	73.4
DNA	/	45.5	83.7	50.7	53.9
Water	19.2	46.3	70.6	44.1	45.4
R.m.s. deviations					
Bond lengths (Å)	0.006	0.008	0.009	0.010	0.008
Bond angles (^{o})	1.16	1.12	1.17	1.31	1.09

[a] Highest resolution shell (in Å) shown in parentheses.
